# Prediction of a Potential Mechanism of Intervertebral Disc Degeneration Based on a Novel Competitive Endogenous RNA Network

**DOI:** 10.1155/2021/6618834

**Published:** 2021-06-30

**Authors:** Junshen Huang, Yuxi Li, Ziwei Ye, Ziying Cheng, Jiajun Huang, Shixin Lu, Kaihui Su, Yuwei Liang, Ming Li, Lin Huang

**Affiliations:** ^1^Department of Orthopedics, Sun Yat-Sen Memorial Hospital, Sun Yat-Sen University, Guangzhou 510120, China; ^2^Institute of Forensic Medicine, West China School of Basic Medical Science & Forensic Medicine, Sichuan University, Chengdu 610065, China

## Abstract

Low back pain which resulted from intervertebral disc degeneration (IDD) is a common health problem that afflicts people all over the world. Due to the lack of an overall understanding of the molecular interactions involved in IDD, we hope to better understand the pathogenetic mechanisms that drive the degenerative process. The purpose of this study is to obtain mRNAs, miRNAs, lncRNAs, and circRNAs associated with IDD gained from public databases and to establish an interaction network. According to the results of microarray analysis and bioinformatics analysis from the contrast of IDD and normal nucleus pulposus tissues, a total of 49 mRNAs, 10 miRNAs, 30 lncRNAs, and 4 circRNAs were obtained and a lncRNA/circRNA–miRNA–mRNA interaction network was constructed. NEAT1–miR-5100–COL10A1 and miR663AHG/HEIH/hsa-circ-0003600–miR-4741–HAS2/HYAL1/LYVE1 might be potential interaction axes of the molecular mechanism in IDD. The increased expression of NEAT1 might inhibit miR-5100 and subsequently upregulate the expression of COL10A1, which leads to IDD, while the increased expression of miR663AHG/HEIH/hsa-circ-0003600 might inhibit miR-4741 and indirectly upregulate HAS2/HYAL1/LYVE1, and leads to the protection from IDD. More interaction axes are to be exploited to provide theoretical bases for further study on IDD.

## 1. Introduction

Low back pain (LBP) is a common health problem that afflicts people all over the world, and it is estimated that around 84% of the world's population will experience low back pain in their lives [1]. Not only does it affect patients' quality of life, but it also has a broad socioeconomic impact, with some recent studies showing that related costs in the United States exceed $100 billion a year [2, 3]. Importantly, intervertebral disc degeneration (IDD) is one of the major risk factors related to LBP [4]. It has been determined that 40% of chronic LBP is caused by IDD [5, 6], and the main factors affecting the pathophysiology of IDD include genetic susceptibility, old age, smoking, alcohol addiction, obesity, and diabetes [7, 8]. Currently, there is no specific treatment for IDD-induced LBP. The current treatment strategies of IDD-induced LBP focus only on pharmacological approaches to relieve pain or inflammation, followed by surgery as a last resort for severe disease stages. With the lack of long-term efficacy and increasing drug abuse, it is critical to develop a molecular targeted therapy for IDD, which may fundamentally prevent IDD or restore the function of the intervertebral disc to solve the problem [9, 10].

IDD is a chronic pathological process with multiple complex etiologies [11]. The intervertebral disc is composed of the inner-most glycosaminoglycan, notochord-derived nucleus pulpous (NP), fibrocartilaginous annulus fibrosus, and superior and inferior cartilaginous end plates. As NP ages and degenerates, it gradually loses its water-bound matrix and becomes fibrotic and its ability to absorb mechanical loads is also diminished, which is accompanied by the phenotype changes in nucleus pulposus cells (NPC) [12]. NPC regulates the balance of synthesis/catabolism by secreting specific biological factors such as CTGF, Shh, and TGF-1, and the secretion changes of these biological factors may also lead to IDD [13]. Understanding the changes of NPC in IDD is essential for the pathogenesis and treatment of the disease.

So far, there have been many reports on the potential influence of IDD genes, such as COL1A1, COL9A2, COL9A3, COL11A2, IL-6, AGC1, VDR, and MMP-3 [14–18]. However, increasing evidences also show that noncoding RNAs (ncRNAs) including miRNA, lncRNA, and circRNA can exert effects on coding RNAs and influence biological processes such as cell proliferation and apoptosis. miRNAs act by binding to complementary sequences in the 3′-untranslated region (UTR) of their target mRNA to trigger transcriptional inhibition or mRNA degradation [19]. lncRNAs and circRNAs can competitively bind to miRNAs through their miRNA reaction elements, thus acting as competitive endogenous RNAs (ceRNAs) to regulate miRNA-target mRNAs' expression levels [20]. More and more studies are revealing the influence of the molecular expression in NPC in the process of IDD disease. However, these results are relatively independent, which cannot provide us a more comprehensive understanding of the molecular expression in the NPC of IDD. Meanwhile, there are still many unknown genes to be explored for a more comprehensive explanation of IDD. There have been few reports on the construction of IDD-related lncRNA/circRNA–miRNA–mRNA networks so far. Therefore, the goal of this study is to filtrate differentially expressed RNAs by using multiple microarray datasets collected from public databases to further construct the regulatory mechanism network of lncRNA/circRNA–miRNA–mRNA in IDD. We aim to identify key molecules in the IDD process and their associated interaction axes, which may provide possible targets for further study.

## 2. Materials and Methods

**Figure 1:**
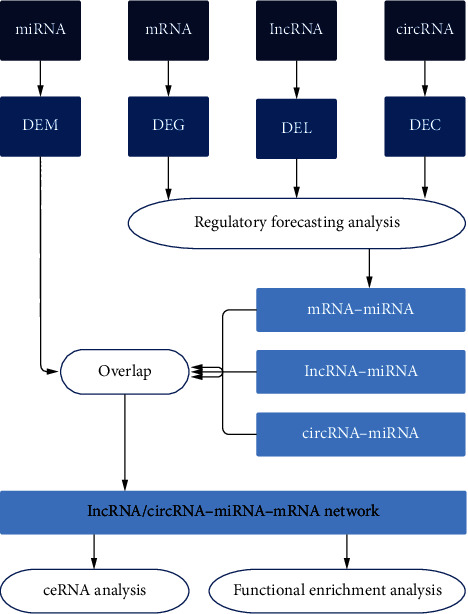
The logical framework is mainly divided into data collection and screening, data analysis, and network construction. The datasets of mRNA, miRNA, lncRNA, and circRNA correlated to IDD were collected from the public database, and DEMs, DEGs, DELs, and DECs with significant expression differences were screened out. Bioinformatics analysis tools and databases were used to conduct regulatory prediction analysis on DEGs, DELs, and DECs and obtain miRNAs paired with them. DEMs was then matched to establish a complete lncRNA/circRNA-miRNA-mRNA network. Then, the network was used to conduct ceRNA analysis and functional enrichment analysis. IDD: intervertebral disc degeneration; DEM: differentially expressed miRNA; DEG: differentially expressed mRNA; DEL: differentially expressed lncRNA; DEC: differentially expressed circRNA; ceRNA: competitive endogenous RNA.

An overall framework of this study, firstly, is shown in Figure 1. The logical framework is mainly divided into “data collection and screening,” “data analysis,” and “network construction”.

### 2.1. Data Collection and Screening

Public functional genomics data was obtained through Gene Expression Omnibus (GEO) (https://www.ncbi.nlm.nih.gov/geo/). To obtain microarray expression datasets for IDD mRNA, miRNA, lncRNA, and circRNA, we used the search keywords “intervertebral Disc” and “Intervertebral Disc” and limited the organism to *Homo sapiens*. By reviewing the summaries, overall designs, and sample sources of these datasets manually, we required that the sample source should be NPC. And in the overall designs, we also required that there were no drug therapy or other interventions in the population to ensure that we could use the selected datasets to screen out the differentially expressed molecules of degenerated NPC.

### 2.2. Data Analysis

Firstly, the differential expression of each dataset was analyzed by using the GEO2R tool [21]. After analysis, the differentially expressed genes (DEGs), differentially expressed miRNAs (DEMs), differentially expressed lncRNAs (DELs), and differentially expressed circRNAs (DECs) between the patients and the control group were obtained. The statistical significance was set to ∣log fold change (logFC) | ≥1, adj. *p* value < 0.05 (raw *p* value was selected if there were too few molecules screened under the condition of adj. *p* value < 0.05). Then, the series matrix files of these datasets were downloaded to do heat map analysis by Morpheus (https://software.broadinstitute.org/morpheus). Intersection analysis was accomplished by the Venn drawing tool of Bioinformatics & Evolutionary Genomics (http://bioinformatics.psb.ugent.be/webtools/Venn/) to gain common differentially expressed RNAs. Besides, functional enrichment analyses on the common DEGs, including Gene Ontology (GO) analysis and Kyoto Encyclopedia of Genes and Genomes (KEGG) analysis, were done by the Database for Annotation, Visualization, and Integrated Discovery (DAVID) v6.8 tool [22, 23].

### 2.3. Protein–Protein Interaction (PPI) Network Construction

In the construction of PPI relationship, we used STRING as the prediction tool. STRING is a known database for predicting protein–protein interactions, including direct physical and indirect functional connections. The interaction relationship is derived from computer prediction, knowledge transfer between organisms, and interactions aggregated from other databases [24]. STRING extract experimental data from numbers of databases like BIND, DIP, and GRID and extract curated data from Biocarta, BioCyc, Reactome, and so on, which guarantee the reliability of mRNA–mRNA relationships in the interaction network. We mapped the DEGs to STRING and validated experimentally only interactions with a combined score > 0.4, which was selected as significant. Hub genes could be found through PPI analysis of common DEGs with the STRING tool, and the PPI network of DEGs was constructed and visualized using Cytoscape software (version 3.6.1; https://www.cytoscape.org.).

### 2.4. miRNA–mRNA Relationship Construction

miRNA-mRNA interactions were established by TargetScan Human 7.2, which is a tool that predicts biological targets of miRNAs by searching for the presence of conserved 8mer, 7mer, and 6mer sites that match the seed region of each miRNA [25]. The target mRNAs associated with DEMs could be predicted using TargetScan Human 7.2. We entered the gene names of each DEMs at TargetScan Human 7.2 to search for their predictive binding mRNAs, and among the predicted results, we selected those with low total context score and high aggregate PCT which suggest high possibility. Then, the target mRNAs were overlapped with the origin DEGs to find the very DEGs that are paired with DEMs and construct the miRNA–mRNA relationship using Cytoscape software.

### 2.5. lncRNA–miRNA Relationship Construction

lncRNA–miRNA relationships were constructed by LncBase Predicted v.2, a tool developed by DIANA Lab which presents an extensive collection of miRNA–lncRNA interactions. This significantly enhanced database includes more than 70000 low- and high-throughput, (in) direct miRNA–lncRNA experimentally supported interactions, derived from manually curated publications and the analysis of 153 AGO CLIP-Seq libraries. LncBase hosts in silico-predicted miRNA targets on lncRNA, identified with the DIANA-microT algorithm [26]. We used LncBase to predict the target lncRNAs related to DEMs, and then, the target lncRNAs were overlapped with the origin DELs to screen the DELs that are paired with DEMs and to construct the lncRNA–miRNA network using Cytoscape software.

### 2.6. circRNA–miRNA Relationship Construction

We used two tools, circBase and RegRNA 2.0, to help construct the circRNA–miRNA relationship. circBase can explore public circRNA datasets and export FASTA files containing genomic sequences [27], while RegRNA 2.0 is a tool that could comprehensively identify the functional RNA motifs and sites in an input RNA sequence, as well as predicting miRNAs that are related to the input circRNA [28]. DEC sequences were obtained from circBase, and then, RegRNA 2.0 was used to predict miRNAs that are related to DECs. After these miRNAs were overlapped with DEMs, DECs paired with DEMs was screened to construct the circRNA–miRNA relationship. The constructed lncRNA–miRNA and circRNA–miRNA relationships were then correlated with the miRNA–mRNA network, and Cytoscape software was used to build the lncRNA/circRNA–miRNA–mRNA network.

## 3. Results

After searching, we obtained a total of 6 mRNA datasets (GSE34095, GSE70362, GSE112216, GSE114169, GSE118927, and GSE124272), 3 miRNA datasets (GSE19943, GSE63492, and GSE116726), 3 lncRNA datasets (GSE56081, GSE112216, and GSE118927), and 1 circRNA dataset, which is GSE67566. The basic information of each dataset is shown in Table 1. Then, by reviewing the summaries, overall designs, and sample sources of these datasets manually, we required that the samples of the selected data sets should be NPC and there were no drug therapy or other interventions in the population in the overall designs.

**Figure 2:**
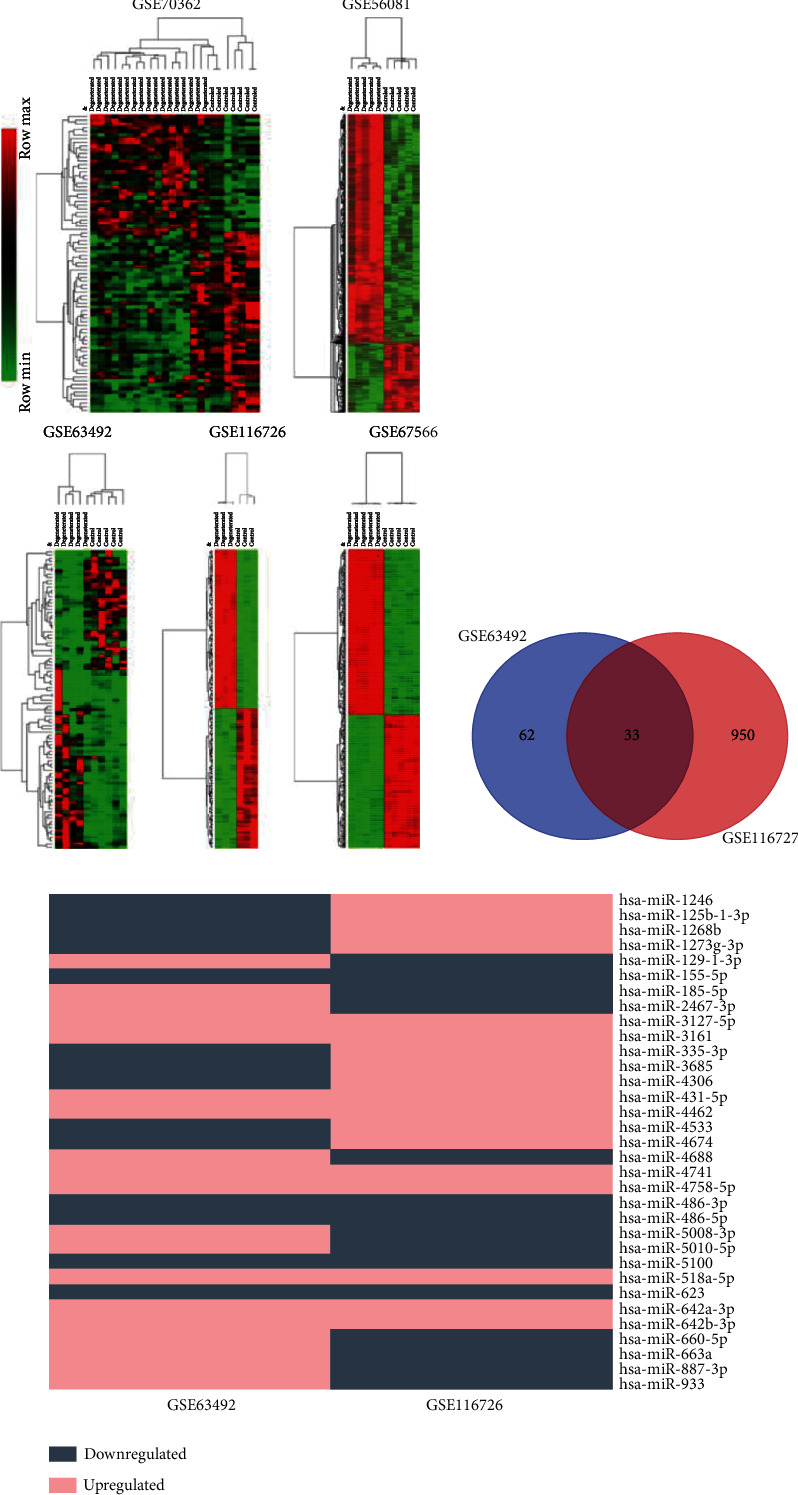
Analysis of differentially expressed RNAs in each dataset. (a) Hierarchical clustering and heat map analysis of differentially expressed RNAs in each dataset. (b) Venn diagram of differentially expressed miRNAs between GSE63492 and GSE116726. (c) A diagram that exhibits the expressions of the 33 miRNAs in the two datasets, and 14 DEMs with consistent expression were ultimately selected, among which 9 were upregulated and 5 were downregulated.

**Figure 3:**
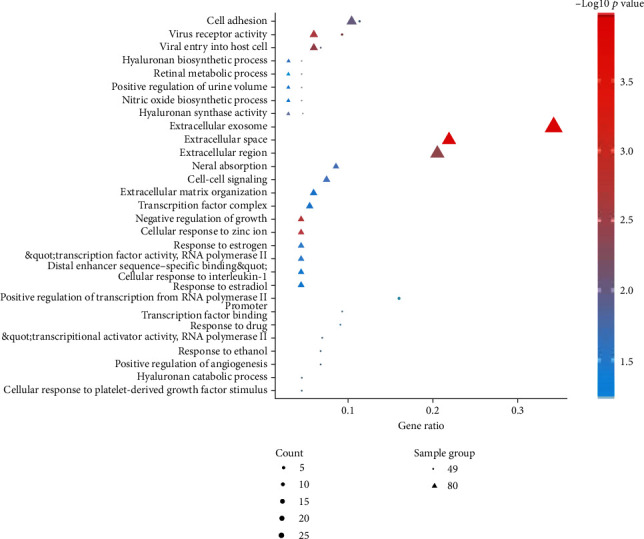
The bubble diagram of gene ontology function enrichment analysis of 80 DEGs and 49 genes that formed the miRNA–mRNA interaction relationship.

It is worth noting that Lan et al. considered that the discs in scoliosis were not strictly normal discs [29], so they reused the normal discs as the control and replaced GSE19943 with GSE63492. Therefore, we also excluded the inclusion of GSE34095. Finally, we selected GSE70362 as the mRNA dataset, GSE63492 and GSE116726 as the miRNA dataset, GSE56081 as the lncRNA dataset, and GSE67566 as the circRNA dataset. According to the preset threshold (adj. *p* value/raw *p* value < 0.05 and ∣logFC | ≥1), we obtained 80 DEGs from GSE70362 (including 32 upregulated and 48 downregulated), 95 DEMs from GSE63492 (including 57 upregulated and 38 downregulated), and 983 DEMs from GSE116726 (including 527 upregulated and 456 downregulated). Besides, 115 DELs were screened out from GSE56081 (containing 50 upregulated and 65 downregulated) and 636 DECs were gained from GSE67566 (consisting of 354 upregulated and 282 downregulated). The top 20 DEGs, DEMs, DELs, and DECs are presented in Tables 2–4. The hierarchical cluster heat maps (Figure 2(a)) indicate that the expression differences of these RNAs were obvious between IDD NPC and normal NPC. Using the Venn drawing tool, the intersection analysis of the two miRNA datasets was performed and 33 DEMs were obtained, as shown in Figure 2(b). The respective expressions of these 33 miRNAs in the two datasets are shown in Figure 2(c), and 14 DEMs with consistent expression were ultimately selected, among which 9 were upregulated and 5 were downregulated. The 80 DEGs were analyzed using DAVID v6.8 to predict their possible biological functions and mechanism pathways. GO enrichment analysis shows 37 terms in total, and Table 5 exhibits 20 of them in which *p* values are under 0.05 (as the *p* values of KEGG analysis results were all greater than 0.05, they were not shown in the table). These DEGs may be involved in biological functions including extracellular exosomes, extracellular space, cellular response to zinc ions, negative regulation of growth, virus receptor activity, and extracellular region. The intuitive display of the enrichment analysis results is shown in Figure 3.

DEM-binding target genes predicted by TargetScan Human 7.2 were compared with 80 DEGs for overlapping. Thus, 10 DEMs and 49 DEGs formed a miRNA–mRNA interaction relationship and GO enrichment analysis was also performed on these 49 DEGs. In Table 5, these DEGs may be involved in the biological processes of the extracellular region, extracellular exosome, transcriptional activator activity, cell inhibiting, transcription factor binding, etc.

**Figure 4:**
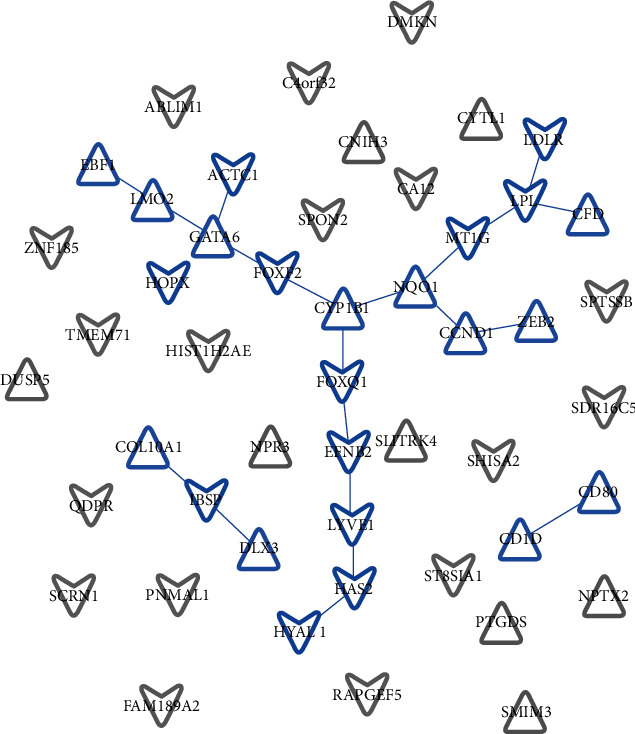
Protein-protein interaction network. Network nodes represent the proteins produced by a single protein-coding gene locus. Blue represents the proteins that jointly contribute to a shared function but do not necessarily mean that they are physically binding each other. Grey represents the proteins without association. Triangle means upregulated expression, whereas arrow means downregulated expression.

The direct display of the enrichment analysis results is shown in Figure 3. A PPI network consisting of 49 nodes and 21 interaction pairs was built using the STRING tool. Among these DEGs, CYP1B1, NQO1, FOXF2, FOXQ1, and GATA6 form an interaction network together, COL10A1, IBSP, and DLX3 form an interaction axis, and CD1D and CD80 form another interaction axis.  Figure 4 shows the protein-protein interaction (PPI) network built by Cytoscape software. Using LncBase Predicted v.2, a total of 5973 lncRNAs were predicted by 10 DEMs, and after eliminating the duplicate data, they were superimposed with 3073 DELs. Thus, the lncRNA–miRNA interaction network between 30 DELs and 8 DEMs was built (11 DELs with downregulated expression regulate 5 DEMs with upregulated expression, while 19 DELs with upregulated expression regulate 3 DEMs).

**Figure 5:**
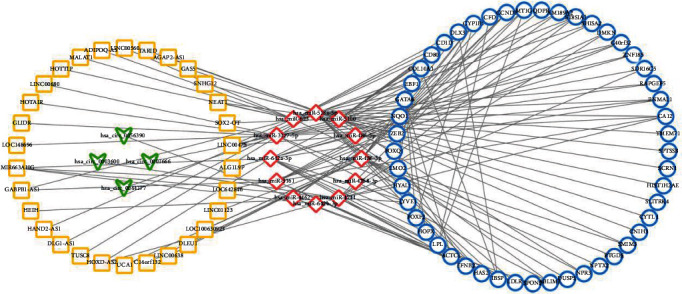
Competing endogenous RNA interaction network of lncRNA/circRNA–miRNA–mRNA. Yellow represents lncRNA, green represents circRNA, red represents miRNA, and blue represents mRNA. Triangle means upregulated expression, whereas arrow means downregulated expression.

Through circBase and RegRNA 2.0 database, 505 miRNAs predicted to interact with DECs were obtained to overlap with 10 DEMs and eventually 4 down-expressed DECs and 3 up-expressed DEMs form an interaction relationship.  Table 6 exhibits the lncRNA/circRNA–miRNA–mRNA relationship and Figure 5 shows the interaction network constructed by Cytoscape software.

## 4. Discussion

As a ubiquitous health problem that affects global health and socioeconomics, it is critical to further study the biological mechanisms behind IDD. Except for the potential IDD mRNA, noncoding RNAs including miRNA, lncRNA, and circRNA may also be the targets used to develop potential therapy strategies. We have reviewed some existing research on the mechanisms of IDD ceRNA. Wang et al. suggested that lncRNA TRPC7-AS1 regulates nucleus pulposus cellular senescence and ECM synthesis via competing with HPN for miR-4769-5p binding [30], and Yang et al. found that lncRNA SLC20A1 promotes extracellular matrix degradation in NP cells by targeting the miR-31-5p/MMP3 axis [31]. In addition, a research suggested that LINC00969 promotes the degeneration of the intervertebral disc by sponging miR-335-3p and regulating NLRP3 inflammasome activation [32]. Except for these researches on lncRNA, there are also literatures reporting the mechanism of circRNA, like circularRNA_104670 can directly bind to miR-17-3p and correct the negative regulation of miR-17-3p on MMP-2, thus inhibiting the apoptosis of NP cells [33]. And hsa_circ_4099 can act as a “sponge” by competitive binding of miR-616-5p, thus reversing the inhibitory effect of miR-616-5p on Sox9 [34]. Noncoding RNA plays an important role in the process of IDD. However, there is still a lack of a comprehensive understanding of the molecular interactions involved in IDD, as there may be other important functional regulation axes. A broad regulatory network of the disease is in need. Based on the existing data from public databases, our study integrated and analyzed the genes and ncRNAs and formed a molecular interaction network. The network we built consists of 49 DEGs, 10 DEMs, 30 DELs, and 4 DECs, as well as containing 375 potential lncRNA/circRNA–miRNA–mRNA axes. We look forward to finding the genes and ncRNAs that are most likely related to IDD through this network, in order to lay a foundation for further research in the future.

**Figure 6:**
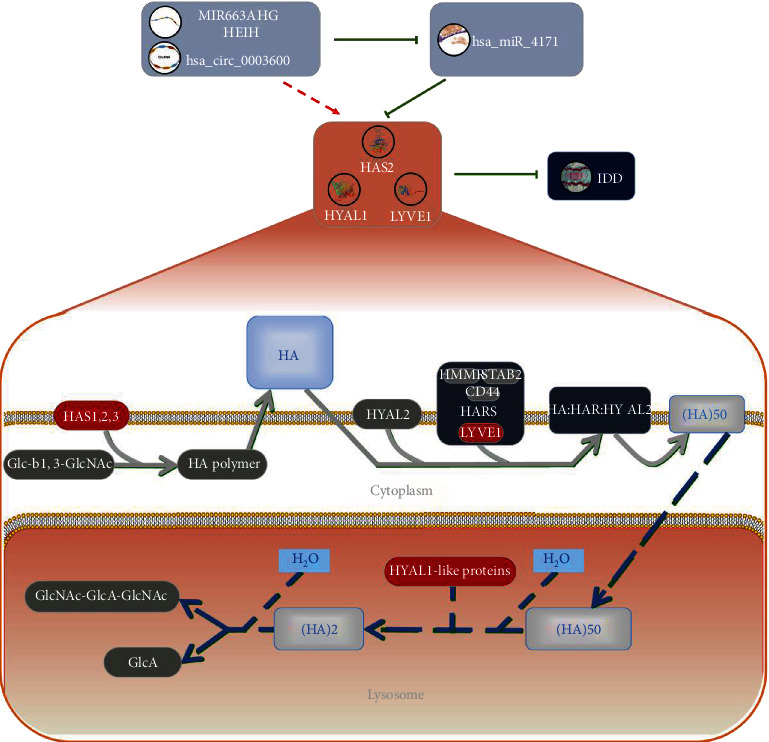
Schematic diagram of hyaluronic acid-related IDD molecular mechanism. The integral membrane dual-action glycosyltransferase proteins hyaluronan synthases 1–3 (HAS1-3) mediate the polymerization of glucuronic acid (GlcA) with N-acetylglucosamine (GlcNAc) to form HA. The resulting polymer has the arrangement [-4GlcA-1,3GlcNAc-]n. LYVE1, HMMR, STAB2, and CD44 together form the HAR complex. High molecular weight HA is tethered to the cell surface by HA receptors and the GPI-linked hyaluronidase 2 (HYAL2) to form a HA : HAR : HYAL2 complex in the plasma membrane that localizes to caveolae, continuing to complete the degradation of HA. In the acidic environment of the lysosome, hyaluronidase 1 (HYAL1) could hydrolyze large 50 disaccharide unit HA fragments to 2 disaccharide units. HAS2, HYAL1, and LYVE1 may be negatively regulated by miR-4741, while mi663ahg/HEIH (lncRNA) and hsa-circ-0003600 (circRNA) may competitively be bound on miR-4741 to act as ceRNAs, correcting the negative regulation of miR-4741 to prevent IDD.

GO enrichment analysis results show that the metabolism of hyaluronan may be one of the important biological processes for IDD. The hyaluronan synthase activity, hyaluronan biosynthetic process, and hyaluronan catabolic process are shown repeatedly in the GO analysis result list. Hyaluronic acid (HA) is one of the major glycosaminoglycan components of the extracellular matrix and is thought to be involved in cell proliferation, migration, and differentiation. HA plays a crucial role in retaining water of the intervertebral disc, so it can provide flexibility and shock absorbance in the spine [35]. Seen from the GO enrichment analysis results, HYAL1, HAS2, and LYVE1 are common gene molecules in hyaluronan-related metabolism. We searched these three genes in the constructed PPI interaction network and found that they constitute the interaction axis of HYAL1–HAS2–LYVE1. These findings suggest that they may indeed participate in the metabolism of hyaluronan and play a common regulatory role in IDD. By reviewing literatures, we found that HAS2 is a member of the gene family that encodes HA synthase, which was elevated in a study of HA-based hydrogel-induced NPC amplification [36]. HYAL1 encodes lysosomal hyaluronidase, which degrades HA in cells. In one study, the gene expression of HYAL1 and the protein expression of HAYL2 were significantly increased in moderate/severe IDD samples compared with those without or with low IDD [37]. The protein encoded by LYVE1 acts as a receptor and binds to both soluble and fixed HA and may also play a role in lymphatic HA transport. It is also been reported in the literature that ingrowth of vascularized fibrous tissue was seen at the edge or within fragments of degenerate disc tissue. Some scattered small vessels were lined by LYVE1+ endothelial cell [38]. In Reactome (https://www.reactome.org), we found a pathway through which HAS2, HYAL1, and LYVE1 coparticipate in HA biological processes (Figure 6). This pathway indicates that the integral membrane dual-action glycosyltransferase proteins hyaluronan synthases 1–3 (HAS1-3) mediate the polymerization of glucuronic acid (GlcA) with N-acetylglucosamine (GlcNAc) to form HA. The resulting polymer has the arrangement [-4GlcA-1,3GlcNAc-]n [39–41]. LYVE1, HMMR, STAB2, and CD44 together form the HAR complex. High molecular weight HA is tethered to the cell surface by HA receptors and the GPI-linked hyaluronidase 2 (HYAL2) to form a HA : HAR : HYAL2 complex in the plasma membrane that localizes to caveolae, continuing to complete the degradation of HA [42–45]. In the acidic environment of the lysosome, hyaluronidase 1 (HYAL1) is able to hydrolyze large 50 disaccharide unit HA fragments to 2 disaccharide units [46]. These theories suggest that the prediction of interaction relationship in this study has certain effectiveness.

In the interaction network constructed in this study, HAS2, HYAL1, and LYVE1 may all be negatively regulated by miR-4741. Meanwhile, mi663ahg/HEIH (lncRNA) and hsa-circ-0003600 (circRNA) may competitively be bound on miR-4741 to act as ceRNAs, correcting the negative regulation of miR-4741. Through literature searching, we found that the mechanism of miR663AHG/HEIH/hsa-circ-0003600 is still lacking in the study of IDD. The interaction axis of miR663AHG/HEIH/hsa-circ-0003600–miR-4741–HAS2/HYAL1/LYVE1 may be one of the potential mechanisms to be studied in IDD.

We also noticed that COL10A1 was the gene with the greatest difference in expression between degenerative NP and normal NP (Table 2), and the results show that it had an aberrant expression in degenerative NP, which is associated with some existing research conclusions. COL10A1 encodes type X collagen, which is specifically expressed and synthesized by hypertrophic chondrocytes, and it plays an important role in the process of cartilage ossification and may be related to matrix degradation, calcification, and vascular invasion [47]. Type X collagen can be localized in combination with advanced intervertebral disc degeneration, and the positive staining of type X collagen is more obvious in the intervertebral disc matrix at the late stage of IDD in elderly patients [48]. In some experiments on mice that have led to disc degeneration, abnormal expression of type X collagen, increased apoptosis, inappropriate intervertebral disc ossification, and elevated expression of COL10A1, Runx2, MMP-13, etc. were observed in degenerative NP [11, 49–51]. Our network analysis also has confirmed that COL10A plays an important role in the pathogenesis of IDD. This not only verifies the credibility of our analysis but also provides an additional theoretical basis for further study on COL10A.

**Figure 7:**
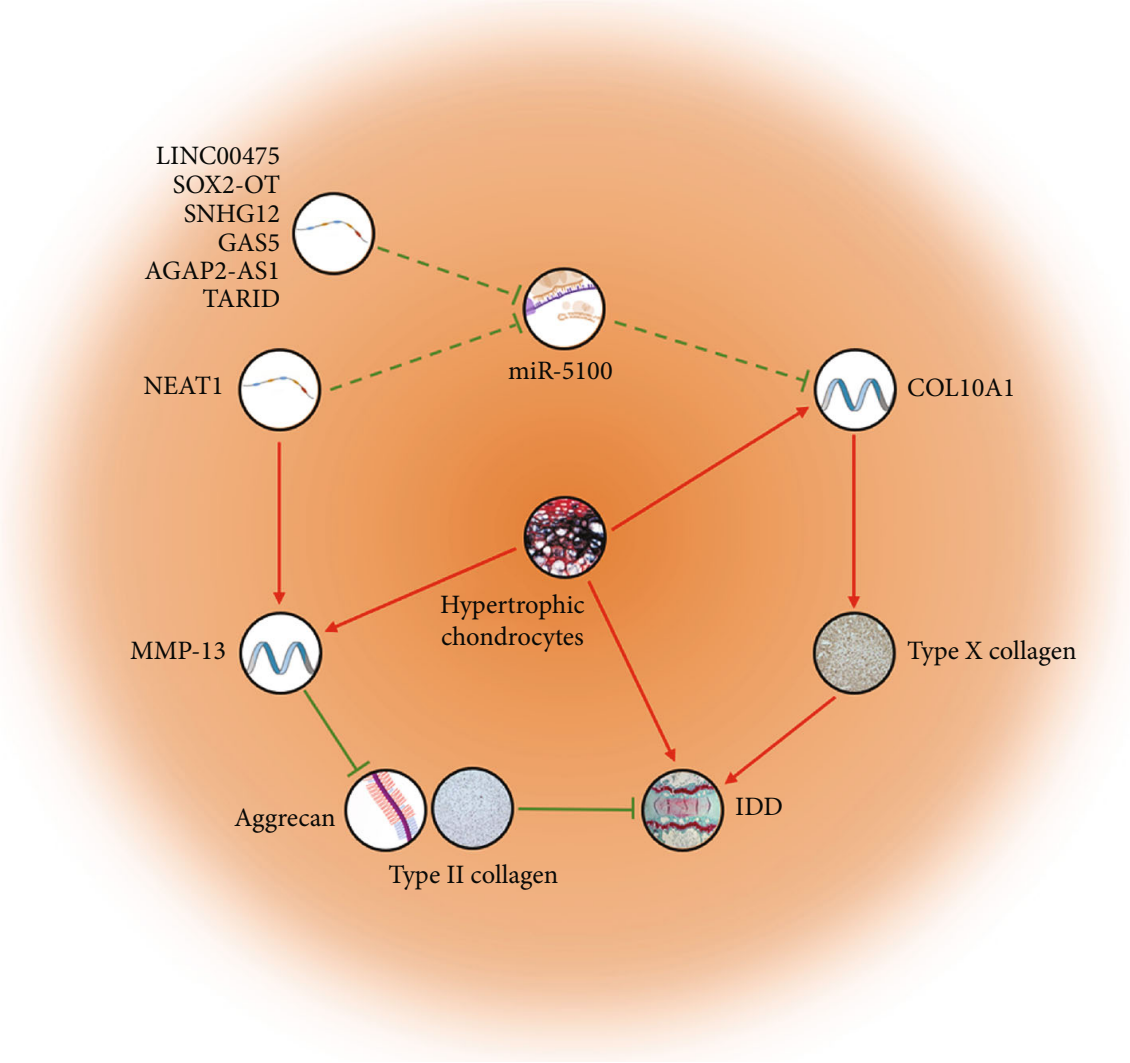
Schematic diagram of COL10A1-related IDD molecular mechanism. The red line represents positive regulation, the green line represents negative regulation, and the arrow represents the direction of regulation. The solid line represents the verified regulatory relationship, and the dashed line represents the regulatory hypothesis proposed in this study. COL10A1 may be negatively regulated by miR-5100, while lncRNA LINC00475, SOX2-OT, NEAT1, SNHG12, GAS5, AGAP2-AS1, and TARID may act as ceRNA for competitively binding miR-5100 and thus correcting the negative regulation of miR-5100, inducing IDD.

In the lncRNA/circRNA–miRNA–mRNA interaction network constructed in this study, COL10A1 may be negatively regulated by miR-5100, while lncRNA LINC00475, SOX2-OT, NEAT1, SNHG12, GAS5, AGAP2-AS1, and TARID may act as ceRNA for competitively binding miR-5100 and thus correcting the negative regulation of miR-5100. Wang et al. verified the contribution of miR-5100 to osteoblast differentiation and mineralization through functional gain and loss experiments, but there is still a lack of studies on miR-5100 in hypertrophic chondrocytes, type X collagen, or IDD. For lncRNA that may negatively regulate miR-5100, the current studies of LINC00475, Sox2-OT, SNHG12, AGAP2-AS1, and TARID are focused on various types of tumours, such as glioma, lymphoma, ovarian cancer, and renal clear cell sarcoma [52–55], while Wang et al. suggested that the overexpression of lncRNA GAS5 might promote the apoptosis of NPC through downregulation of Bcl-2 and upregulation of caspase-3 [56]. It is worth noting that NEAT1 may be the ceRNA-regulating COL10A1. Ruan et al. suggest that NEAT1 has been demonstrated to participate in ECM remodelling and they found the that increased expression of NEAT1 decreased aggrecan and type II collagen levels and increased ADAMTS4 and MMP-13 levels [57]. Our analysis results showed that NEAT1 expression was increased in IDD patients (logFC = 1.78), further confirming that NEAT1 may play an important role in IDD. Some research has proved that a robust expression of type X collagen and MMP-13 mRNA and protein could occur by hypertrophic chondrocytes during IDD [51, 58]. This suggests that NEAT1 and COL10A1 are connected by type X collagen and MMP-13. The ceRNA network shows that both NEAT1 and COL10A1 can interact with miR-5100 to form the interaction axis. Thus, we build a schematic diagram of the IDD molecular mechanism related to NEAT1 and COL10A1 (Figure 7). Although it can be hypothesized that in IDD patients, the NEAT1–miR-5100–COL10A1 interaction axis may be unbalanced, leading to the occurrence of IDD; there is still a lack of evidence that NEAT1 can negatively regulate miR-5100 and thereby regulate COL10A1 to realize ECM remodelling. Further elucidation is needed to confirm this hypothesis in the future.

## 5. Conclusions

Through bioinformatics analysis of multiple datasets, we constructed a ceRNA interaction network of lncRNA/circRNA–miRNA–mRNA for IDD. The result of this study verifies that the process of cartilage ossification, matrix degradation, and calcification following the synthesis of type X collagen and the imbalance of hyaluronic acid metabolism are two explanations for the occurrence of IDD. NEAT1–miR-5100–COL10A1 and miR663AHG/HEIH/hsa-circ-0003600–miR-4741–HAS2/HYAL1/LYVE1 might be potential interaction axes of the molecular mechanism in IDD. The increased expression of NEAT1 might inhibit miR-5100 and subsequently upregulate the expression of COL10A1, which leads to IDD, while the increased expression of miR663AHG/HEIH/hsa-circ-0003600 might inhibit miR-4741 and indirectly upregulate HAS2/HYAL1/LYVE1, and leads to the protection from IDD. This novel ceRNA network could be used to predict potential mechanisms to reach a comprehensive understanding and could provide possible targets for the further study of IDD in the future.

## Figures and Tables

**Table 1:** The basic information of the datasets obtained from GEO database.

Dataset	Type of RNAs tested	Samples	Experimental group	Control group	Platform
GSE34095	mRNA	6	Degenerative nucleus pulposus from IDD patients	Nucleus pulposus from scoliosis patients	GPL96 [HG-U133A] Affymetrix Human Genome U133A Array
GSE70362	mRNA	24	Degenerative nucleus pulposus (Thompson grade III–V)	Degenerative nucleus pulposus (Thompson grade I–II)	GPL17810 [HG-U133_Plus_2] Affymetrix Human Genome U133 Plus 2.0 Array [CDF: Brainarray HGU133Plus2_Hs_ENTREZG_v16]
GSE112216	mRNA, lncRNA	6	Degenerative nucleus pulposus derived cells cocultured with adipose derived stem cell	Degenerative nucleus pulposus derived cells	GPL16686 [HuGene-2_0-st] Affymetrix Human Gene 2.0 ST Array [transcript (gene) version]
GSE114169	mRNA	8	Degenerative nucleus pulposus cultured 1 week with Neurotropin	Degenerative nucleus pulposus cultured 1 week with none	GPL17077 Agilent-039494 SurePrint G3 Human GE v2 8x60K Microarray 039381 (Probe Name version)
GSE118927	mRNA, lncRNA	6	Degenerative nucleus pulposus derived cells cocultured with adipose derived stem cell	Degenerative nucleus pulposus derived cells	GPL16686 [HuGene-2_0-st] Affymetrix Human Gene 2.0 ST Array [transcript (gene) version]
GSE124272	mRNA	16	Peripheral blood from IDD patients	Peripheral blood from healthy people	GPL21185 Agilent-072363 SurePrint G3 Human GE v3 8x60K Microarray 039494 [Probe Name version]
GSE19943	miRNA	6	Degenerative nucleus pulposus from IDD patients	Nucleus pulposus from scoliosis patients	GPL9946 Exiqon human miRCURY LNA™ microRNA Array V11.0
GSE63492	miRNA	10	Degenerative nucleus pulposus	Nondegenerative nucleus pulposus	GPL19449 Exiqon miRCURY LNA microRNA Array; 7th generation REV-hsa; mmu & rno (miRBase v18.0)
GSE116726	miRNA	6	Degenerative nucleus pulposus from IDD patients	Nucleus pulposus from traumatic lumbar fracture	GPL20712 Agilent-070156 Human miRNA [miRNA version]
GSE56081	lncRNA	10	Degenerative nucleus pulposus	Nondegenerative nucleus pulposus	GPL15314 Arraystar Human LncRNA microarray V2.0 (Agilent_033010 Probe Name version)
GSE67566	circRNA	10	Degenerative nucleus pulposus	Nondegenerative nucleus pulposus	GPL19978 Agilent-069978 Arraystar Human CircRNA microarray V1

**Table 2:** Top 20 upregulated and downregulated differentially expressed mRNAs.

GSE70362					
DEG	*p* value	logFC	DEG	*p* value	logFC
COL10A1	0.017000	2.32	HIST1H2AE	0.036200	−1.48
IGFBP3	0.007980	2.24	Scgb2a2	0.008330	−1.50
SMIM3	0.003260	1.78	Mt1m	0.012000	−1.50
LMO2	0.000055	1.75	FOXQ1	0.000920	−1.61
GDF15	0.000670	1.73	MT1G	0.002230	−1.79
TFPI	0.002200	1.63	SCGB1D2	0.026100	−1.83
CCND1	0.000055	1.60	HOPX	0.001200	−1.86
NPTX2	0.032000	1.55	KRT19	0.009330	−1.92
EMILIN1	0.000110	1.51	SDR16C5	0.000174	−1.92
TNFAIP6	0.002250	1.43	IBSP	0.005660	−2.22

DEG: differentially expressed mRNA.

**Table 3:** Top 20 upregulated and downregulated differentially expressed miRNAs.

GSE63492			GSE116726		
DEM	*p* value	logFC	DEM	Adj. *p* value	logFC
hsa-miR-4287	0.004497	5.81	hsa-miR-29c-3p	0.000033	2.58
hsa-miR-3150a-3p	0.000012	5.03	hsa-miR-187-3p	0.000001	2.52
hsa-miR-3157-3p	0.010912	4.90	hsa-miR-141-3p	0.000001	2.52
hsa-miR-660-5p	0.00199	4.80	hsa-miR-455-5p	0.000009	2.52
hsa-miR-887-3p	0.000105	4.05	hsa-miR-34a-5p	0.000065	2.46
hsa-miR-5010-5p	0.026553	4.03	hsa-miR-610	0.000079	2.44
hsa-miR-933	0.00739	3.43	hsa-miR-139-5p	0.000378	2.42
hsa-miR-3127-5p	0.004452	3.35	hsa-miR-223-5p	0.000001	2.41
hsa-miR-4450	0.002228	3.24	hsa-miR-338-3p	0.000008	2.41
hsa-miR-516a-5p	0.000229	3.15	hsa-miR-127-5p	0.000001	2.40
hsa-miR-193a-5p	0.01867	−2.65	hsa-miR-6857-5p	0.000999	−1.74
hsa-miR-1246	0.036306	−2.92	hsa-miR-3654	0.000581	−1.85
hsa-miR-4454	0.017803	−2.97	hsa-miR-204-5p	0.00703	−2.26
hsa-miR-4327	0.004021	−3.02	hsa-miR-590-5p	0.000082	−2.34
hsa-miR-155-5p	0.017538	−3.22	hsa-miR-181c-5p	0.000292	−2.40
hsa-miR-196b-5p	0.013547	−3.43	hsa-miR-378a-5p	0.000002	−2.46
hsa-miR-3648	0.000614	−3.76	hsa-miR-410-5p	0.000001	−2.49
hsa-miR-486-3p	0.008002	−4.39	hsa-miR-376a-5p	0.000001	−2.50
hsa-miR-125b-1-3p	0.000444	−4.69	hsa-miR-486-5p	0.000003	−2.77
hsa-miR-1184	0.000544	−4.85	hsa-miR-32-5p	0.000001	−2.81

DEM: differentially expressed miRNA.

**Table 4:** Top 20 upregulated and downregulated differentially expressed lncRNAs and circRNAs.

GSE56081			GSE67566		
DEL	Adj. *p* value	logFC	DEC	Adj. *p* value	logFC
TRPC7-AS1	6.55*E*-08	6.61	hsa_circRNA_101852	3.92*E*-15	2.98
MIR4458HG	5.56*E*-03	1.40	hsa_circRNA_101853	6.98*E*-16	2.93
GAS5	4.30*E*-02	1.40	hsa_circRNA_101139	6.98*E*-16	2.92
CBR3-AS1	4.30*E*-04	1.40	hsa_circRNA_103890	1.72*E*-15	2.86
ADPGK-AS1	2.67*E*-03	1.40	hsa_circRNA_400019	3.87*E*-14	2.84
SNHG5	2.67*E*-02	1.40	hsa_circRNA_102324	1.00*E*-15	2.78
ADARB2-AS1	4.17*E*-03	1.39	hsa_circRNA_104703	1.24*E*-15	2.72
LINC00431	4.68*E*-04	1.39	hsa_circRNA_104600	7.51*E*-15	2.68
MCCC1-AS1	8.89*E*-03	1.39	hsa_circRNA_100604	1.57*E*-15	2.68
MALAT1	6.58*E*-04	1.07	hsa_circRNA_100018	1.67*E*-15	2.61
LINC01405	9.52*E*-04	−3.04	hsa_circRNA_105031	1.18*E*-14	−2.83
LINC00884	5.11*E*-05	−3.12	hsa_circRNA_101370	1.82*E*-14	−2.86
HAND2-AS1	3.47*E*-04	−3.21	hsa_circRNA_104019	2.04*E*-13	−2.97
EFCAB6-AS1	2.96*E*-08	−3.39	hsa_circRNA_104630	2.41*E*-14	−3.04
LINC00689	2.87*E*-05	−3.63	hsa_circRNA_101709	1.18*E*-14	−3.04
MAPT-AS1	6.53*E*-09	−5.12	hsa_circRNA_101557	1.96*E*-14	−.05
IL10RB-AS1	9.48*E*-09	−5.54	hsa_circRNA_103838	6.98*E*-16	−3.06
VPS13A-AS1	1.81*E*-08	−6.01	hsa_circRNA_102116	1.92*E*-14	−3.18
LINC00957	7.33*E*-09	−6.28	hsa_circRNA_104508	2.19*E*-13	−3.26
HOTAIR	6.47*E*-08	−7.21	hsa_circRNA_101645	1.60*E*-14	−3.30

DEL: differentially expressed lncRNA; DEC: differentially expressed circRNA.

**Table 5:** Gene ontology enrichment analysis of 80 DEGs and 49 genes that formed the miRNA–mRNA interaction relationship.

Sample group	Category	Term	Count	*p* value	Genes
80	CC	Extracellular exosome	25	0.000123351	GALNT3; LPL; HYAL1; ACTC1; HIST1H2AE; QDPR; SELENBP1; NPR3; TMEM27; EMILIN1; LYVE1; KRT19; TEX14; PTGDS; VAMP8; DMKN; AOX1; GDF15; CFD; SPON2; NQO1; DEFB1; IGFBP3; DCXR; HIST1H4H
80	CC	Extracellular space	16	0.000207983	IBSP; HYAL1; LPL; ACTC1; CYTL1; SELENBP1; PTHLH; TNFAIP6; PTGDS; SCGB1D2; TFPI; SPON2; CFD; GDF15; IGFBP3; DEFB1
49	MF	Virus receptor activity	4	0.00065	HYAL1; LDLR; CD80; EFNB2
80	BP	Cellular response to zinc ion	3	0.002491898	MT1M; MT1G; MT1F
80	BP	Negative regulation of growth	3	0.002491898	MT1M; MT1G; MT1F
80	MF	Virus receptor activity	4	0.002592014	HYAL1; LDLR; CD80; EFNB2
80	CC	Extracellular region	15	0.003781513	IBSP; LPL; EMILIN1; PTHLH; PTGDS; NPTX2; DMKN; TFPI; TREM1; CFD; GDF15; IGFBP3; DEFB1; HIST1H4H; COL10A1
80	BP	Viral entry into host cell	4	0.003840079	LDLR; CD80; VAMP8; EFNB2
80	BP	Cell adhesion	7	0.009200697	IBSP; TNFAIP6; LYVE1; CYP1B1; EFNB2; SPON2; EMILIN1
49	MF	Hyaluronan synthase activity	2	0.009680585	HYAL1; HAS2
80	MF	Hyaluronan synthase activity	2	0.015548781	HYAL1; HAS2
49	BP	Viral entry into host cell	3	0.017061685	LDLR; CD80; EFNB2
49	BP	Hyaluronan biosynthetic process	2	0.017380591	HYAL1; HAS2
80	BP	Cell-cell signaling	5	0.017725805	PTHLH; TNFAIP6; CD80; EFNB2; GDF15
80	TH	Neral absorption	3	0.019704118	MT1M; MT1G; MT1F
49	MF	Transcriptional activator activity, RNA polymerase II transcription regulatory region sequence spec	3	0.022287725	LMO2; GATA6; FOXF2
80	BP	Response to estrogen	3	0.026997667	KRT19; CCND1; GATA6
80	BP	Hyaluronan biosynthetic process	2	0.027195604	HYAL1; HAS2
49	BP	Cell adhesion	5	0.027213497	IBSP; LYVE1; CYP1B1; EFNB2; SPON2
80	MF	Transcription factor activity, RNA polymerase II distal enhancer sequence-specific binding	3	0.027502988	T; GATA6; FOXF2
49	BP	Response to ethanol	3	0.028347603	ACTC1; CCND1; NQO1
49	BP	Retinal metabolic process	2	0.029614391	CYP1B1; SDR16C5
49	MF	Transcription factor binding	4	0.031391824	HYAL1; CCND1; GATA6; FOXF2
80	BP	Cellular response to interleukin-1	3	0.031774689	HYAL1; TFPI; HAS2
49	BP	Positive regulation of urine volume	2	0.032043246	HAS2; NPR3
49	BP	Nitric oxide biosynthetic process	2	0.032043246	CYP1B1; NQO1
49	BP	Positive regulation of angiogenesis	3	0.033505165	HYAL1; CYP1B1; GATA6
49	BP	Positive regulation of transcription from RNA polymerase II promoter	7	0.033985253	DLX3; LMO2; GATA6; EBF1; FOXF2; CYTL1; ZEB2
49	BP	Hyaluronan catabolic process	2	0.036883166	HYAL1; LYVE1
49	BP	Response to drug	4	0.040149506	LPL; ACTC1; CCND1; GATA6
80	CC	Transcription factor complex	4	0.040958701	LMO2; GATA6; FOXF2; PDLIM1
80	BP	Extracellular matrix organization	4	0.041931952	IBSP; FOXF2; EMILIN1; COL10A1
49	BP	Cellular response to platelet-derived growth factor stimulus	2	0.044098781	HYAL1; HAS2
80	BP	Retinal metabolic process	2	0.046173764	CYP1B1; SDR16C5
80	BP	Response to estradiol	3	0.049819454	CCND1; TFPI; NQO1
80	BP	Nitric oxide biosynthetic process	2	0.049925405	CYP1B1; NQO1
80	BP	Positive regulation of urine volume	2	0.049925405	HAS2; NPR3

MF: molecular function; BP: biological process; CC: cellular component.

**Table 6:** lncRNA/circRNA–miRNA–mRNA relationship.

DEM	DEG	DEL	DEC
hsa-miR-3127-5p	CA12; HYAL1; LYVE1; PNMAL1; RAPGEF5; SDR16C5; ZNF185	LINC00680; HOTAIR; GLIDR; LOC148696	hsa_circ_0044177; hsa_circ_0001666
hsa-miR-3161	ACTC1; C4orf32; CA12; DMKN; EFNB2; HAS2; IBSP; SHISA2; ST8SIA1	MIR663AHG; GABPB1-AS1	—
hsa-miR-4462	FAM189A2; LDLR; QDPR; SPON2	—	hsa_circ_0056390
hsa-miR-4741	CA12; DMKN; HAS2; HYAL1; LDLR; LYVE1; MT1G; SDR16C5; ST8SIA1	MIR663AHG; HEIH	hsa_circ_0003600
hsa-miR-4758-5p	ABLIM1	—	—
hsa-miR-518a-5p	ABLIM1; C4orf32; CA12; EFNB2; FOXQ1; HIST1H2AE; IBSP; LDLR; LYVE1; PNMAL1; RAPGEF5; SCRN1; SHISA2; SPTSSB; ST8SIA1; TMEM71	GABPB1-AS1; HAND2-AS1; DLG1-AS1; TUSC8	—
hsa-miR-642a-3p	C4orf32; EFNB2; FAM189A2; FOXF2; HOPX; LPL; LYVE1; PNMAL1; ZNF185	MIR663AHG; HOXD-AS2	—
hsa-miR-486-3p	CCND1; CFD; CYP1B1; DLX3; DUSP5; NPR3; NPTX2; PTGDS; SMIM3	UCA1; C14orf132; LINC00638; DLEU1; LOC100630923; LINC01123; LOC642846	—
hsa-miR-5100	CD1D; CD80; CFD; CNIH3; COL10A1; CYP1B1; CYTL1; EBF1; GATA6; NPR3; NQO1; SLITRK4; SMIM3; ZEB2	LINC00475; SOX2-OT; NEAT1; SNHG12; GAS5; AGAP2-AS1; TARID	—
hsa-miR-623	CCND1; CD80; CYP1B1; EBF1; GATA6; LMO2	NEAT1; LINC00560; ADIPOQ-AS1; MALAT1; HOTTIP; LINC00475	—

## Data Availability

All data generated or analyzed during this study are included in this published article. The datasets analyzed during the current study are available in the Gene Expression Omnibus (GEO) (https://www.ncbi.nlm.nih.gov/geo/).
